# Effect of the infection with the nematode *Haemonchus contortus* (Strongylida: Trichostrongylidae) on the haematological, biochemical, clinical and reproductive traits in rams

**DOI:** 10.4102/ojvr.v83i1.1129

**Published:** 2016-08-30

**Authors:** Mariem Rouatbi, Mohamed Gharbi, Mohamed R. Rjeibi, Imen Ben Salem, Hafidh Akkari, Narjess Lassoued, Mourad Rekik

**Affiliations:** 1 Laboratory of Parasitology, Manouba University, Tunisia; 2 Department of Animal Production, Service of Animal Science, Manouba University, Tunisia; 3 Department of Animal and Forage, National Institute of Agronomic Research of Tunisia, Tunisia; 4 International Center for Agricultural Research in the Dry Areas, Amman, Jordan

## Abstract

This study aimed to investigate the effect of *Haemonchus contortus* infection on rams’ haematological, biochemical and clinical parameters and reproductive performances. A total number of 12 Barbarine rams (control and infected) were included in the experiment. The infected group received 30 000 *H. contortus* third-stage larvae orally. Each ram’s ejaculate was immediately evaluated for volume, sperm cell concentration and mortality rate. At the end of the experiment (day 82 post-infection), which lasted 89 days, serial blood samples were collected in order to assess plasma testosterone and luteinising hormone (LH) concentrations. There was an effect of time, infection and their interaction on haematological parameters (*p* < 0.001). In infected rams, haematocrit, red blood cell count and haemoglobin started to decrease from 21 days post-infection. There was an effect of time and infection for albumin. For total protein, only infection had a statistically significant effect. For glucose, only time had a statistically significant effect. Concentrations were significantly lower in infected rams compared to control animals. A significant effect of infection and time on sperm concentrations and sperm mortality was observed. The effect of infection appears in time for sperm concentrations at days 69 and 76 post-infection. Sperm mortality rate was significantly higher in infected animals at day 46 post-infection when compared to control group (*p* < 0.05). Finally, plasma testosterone traits (average concentration, cumulated levels during the sampling period and pulse frequency) were depressed in infected rams when compared to control counterparts; none of these endocrine traits were affected for plasma LH.

## Introduction

Thermo-period, photoperiod and nutrition are usually regarded as the main environmental factors affecting reproductive performance of livestock species under arid and semi-arid environments (Gonzalez-Bulnes *et al*. 2010). In small ruminants, the impact of disease on reproductive efficiency is not as well studied as in cattle and particularly when it is in relation to enzootic diseases such as gastrointestinal helminth infections. In Southern Mediterranean countries, small ruminants face important health problems with high prevalences of several diseases such as brucellosis, foot and mouth disease, border disease and several parasitic infections such as *Toxoplasma gondii* infection (Gharbi *et al*. 2013), fasciolosis (Akkari, Gharbi & Darghouth 2011), lungworms and gastrointestinal helminths (Akkari, Gharbi & Darghouth 2012).

In Tunisia, for example, control measures of gastrointestinal helminths are hampered by (1) a very important diversity of epidemiological features through a north–south axis going, respectively, from humid to Saharan climate (Darghouth, unpublished data), (2) the absence of standard control programmes against gastrointestinal helminths based on epidemiological data and (3) very high parasite infection indicators. Indeed, Akkari *et al*. (2012) showed that 45.5% of the parasite population were found in the abomasum, with the prevalence of *Haemonchus contortus* exceeding 35%.

While the hematophagous nature of this common pathogenic parasite is well documented, there is little information on how essential functions such as reproduction may be affected when animals are infected with *H. contortus*. It is commonly assumed that, intuitively, parasite infection renders the animals ill, hence affecting all functions. Nevertheless, chronically affected animals are commonly present in flocks kept for production and reproduction without any objective assessment of the shortfalls on their reproductive performance. This study therefore attempted to study the effects of a chronic infection with *H. contortus* on semen and associated endocrine traits of adult Barbarine rams.

## Materials and methods

The trial was carried out during the transition phase between the breeding and the non-breeding seasons (from November to February). The work was performed in the governorate of Ben Arous, at the experimental station of Bourebiaà (National Institute of Agronomic Research of Tunisia [INRAT]; latitude 36° 38 N; longitude 10° 07 E). The average annual rainfall (the mean of the last 30 years) was 350 mm. Averages of the ambient temperature were 10.6 °C, 9.4 °C and 10.3 °C during December, January and February, respectively. Ethical concerns were taken into account by adhering to local animal welfare regulations and practices and conformed to ethical guidelines for animal usage in research of the National School of Veterinary Medicine of Sidi Thabet (Tunisia) and the Association Tunisienne des Sciences des Animaux de Laboratoire (ATSAL/0116, Tunisia).

### Animals and management

A total number of 12 healthy adult Barbarine rams accustomed to ejaculation in an artificial vagina, aged between 2.5 and 5 years (mean: 3.25 years), weighing between 46 kg and 71 kg (mean: 62 kg) were included in the trial. The choice of the number of animals was motivated by the principle of reduction proposed by Russell and Burch (1959). They were divided into two homogenous groups (infected and control) which did not statistically differ in age and live weight. The rams were housed individually in cleaned experimental pens of 2 m^2^ (1 m × 2 m), and they were allowed an adaptation period of 18 days for the new housing and feeding conditions; they were fed *ad libitum* with hay and had continuous access to fresh water. Throughout the experiment, the animals were exposed to natural daylight.

### Parasitic infection and experimental treatments

All the animals were initially drenched with albendazole (Dalben^®^ 1.9, CEVA laboratories, Tunis, Tunisia) (7 mg/kg) two times at an interval of three days. The animals were sampled for coprology with the flotation and Baermann (Bussiéras & Chermette 1995; Euzéby 1981) techniques every week for three consecutive weeks. During the third week, all faecal samples were negative for both respiratory and gastrointestinal helminths. The rams belonging to the infected group received 30 000 *H. contortus* third-stage larvae orally as described by Bordoloi, Jas and Ghosh (2012). *Haemonchus contortus* larvae were obtained from a donor ram infected with this parasite. This was considered as the starting point of the experiment which lasted for 89 days. Care was taken for control animals not to be in contact, directly or indirectly, with infected animals. For everyday care and for experimental interventions, workers and staff always started with control animals before moving to the infected ones.

### Parasitological, haematological, biochemical and clinical parameters

On a weekly basis, throughout the experiment and for all animals, faecal egg counts (FEC) were qualitatively assessed to verify whether gastrointestinal parasite eggs were present or absent in the faeces. For positive rams, the quantification was done on day 30 and day 70 post-infection with McMaster technique (Raynaud 1970) consisting of two counting chambers. The formula that was used is FEC = number of eggs in the two compartments × 50. EDTA blood samples were collected weekly from the rams. Plasma was recovered and stored at -20 °C until analysed. Haematological parameters: haematocrit (%), red blood cell count (×10^6^/mL) and haemoglobin (g/dL) were estimated using an Auto Haematology analyser BC-2800Vet^®^ (Shenzen Mindray Bio-Medical Electronics Co., Ltd, Hamburg, Germany). Plasma samples were also used to estimate biochemical indicators: albumin (g/L), glucose (mmol/L) and total proteins (g/L) with a Random Access Clinical Autolyzer^®^ (Dialab, Vienna, Austria). Heart rate (beats per minute), respiratory rate (breaths per minute) and rectal temperature (°C) were measured weekly.

### Live weight and scrotal circumference

Live weight and scrotal circumference were measured every 2 weeks. At each occasion, the rams were weighed twice before being fed in the morning. Scrotal circumference was assessed with a measuring tape (± 1 mm) at the greatest diameter along the scrotum with the ram in a standing position. No correction was made for scrotal skin thickness.

### Semen collection and evaluation

Starting 25 days after infection with *H. contortus* larvae, the volume of ejaculate, sperm concentration and sperm mortality rate were measured at weekly intervals by collecting semen using an artificial vagina kept in a water bath at 35 °C. During semen collection, animals were put individually in the presence of a teaser female that was previously induced into oestrus by inserting a progestagen-impregnated vaginal sponge for 10 days. Each ejaculate was recovered in glass tubes graduated to the nearest 0.1 mL allowing determination of the volume directly in the tube. Concentration of spermatozoids (number of spermatozoa/mL) was estimated after diluting semen in 9‰ sodium chloride using a spectrophotometer at 550 nm wave length (Accucell^®^, IMV, L’Aigle, France). Assessment of dead spermatozoa was undertaken utilising an eosin–nigrosin staining method described by Baril *et al*. (1993).

### Plasma testosterone and luteinising hormone concentrations

Eighty-two days post-infection, serial blood samples were taken during six consecutive hours every 20 min from the jugular vein with heparinised vacutainer^®^ tubes. Plasma was collected after being centrifuged (3000 g, 15 min), then stored at -20 °C until assayed. Plasma concentrations of testosterone and luteinising hormone (LH) were estimated using a monoclonal antibody, non-extraction radioimmunoassay method. Plasma testosterone and LH concentrations were determined in duplicate with Coat-A-Count^®^ radioimmunoassay kits (Diagnostic Products Corporation, Los Angeles, USA), according to the manufacturer’s instructions. For both testosterone and LH, all samples were, respectively, included in one single assay. Intra-assay variation coefficients were 2.51% and 10.89%, respectively, for testosterone and LH concentrations. An increase greater than the mean of testosterone (or LH) in all samples taken in the 6-h period plus two standard deviations followed by a decrease was considered as a pulse (Diekman *et al*. 1991).

### Statistical analyses

Results for haematocrit, red blood cell count, haemoglobin, albumin, total proteins, glucose, live weight, scrotal circumference, concentration, sperm mortality rate in the ejaculates and the ejaculates volume were analysed using factorial Analysis of variance (ANOVA) with two independent factors: infection with *H. contortus* and time. The analysis was performed using SPSS software for Windows version 18. These analyses allow testing the effect of treatment, time and their interaction. When treatment and time had a significant effect, further analyses were performed using repeated-measures analysis of variance with XlSTAT^®^, adware for Excel^®^ to depict when this effect appears over time. For individual profiles of testosterone and LH, areas delineated by measured concentrations were calculated using the trapezoid rule using GeoGebra^®^ (version 4.0, International GeoGebra Institute, Linz, Australia). Data on the number of testosterone pulses frequency were converted to the square root of *n* + 0.5 because some rams, particularly in the control group, had zero pulse frequencies (Dickson & Sanford 2005). The comparison between groups of animals was undertaken using Student’s *t*-test. The Pearson’s correlation was used to assess the relationship between testosterone and LH plasma concentrations. The effect of infection was considered significant when the level of probability was 0.05 or less. Results are presented as means ± S.E.M unless specified otherwise.

Threshold values used for haematological and biochemical parameters are shown in Table 1 (Blood & Radostits 1989).

**TABLE 1:** Haematological and biochemical threshold values.

Parameter	Threshold value
**Haematological parameters**	
Red blood cells (10^12^/L)	5.0–14.0
Haemoglobin (g/dL)	9.0–15.5
Haematocrit (%)	26.0–45.0
**Biochemical parameters**	
Albumin (g/L)	24–30
Glucose (mmol/L)	2.8–4.5
Total proteins (g/L)	60–79

*Source*: Blood, D.C. & Radostits, O.M., 1989, *Veterinary medicine*, Baillière Tindall, London

## Results

### Parasitological infection

Faecal samples of all animals were negative for gastrointestinal and lungworms immediately before infection. Three weeks after infection, infected rams started shedding *H. contortus* eggs while none of the control rams excreted eggs. This worm burden was maintained throughout the experiment (to day 89) and was weekly verified by qualitative coprology. Infected rams excreted *H. contortus* eggs throughout the experiment but none of the control animals did. At days 30 and 70 post-infection, the faecal egg counts in the six infected animals reached an average of 6200 eggs/g ± 664.24 eggs/g and 5000 eggs/g ± 886.00 eggs/g, respectively.

### Haematological parameters

**FIGURE 1:**
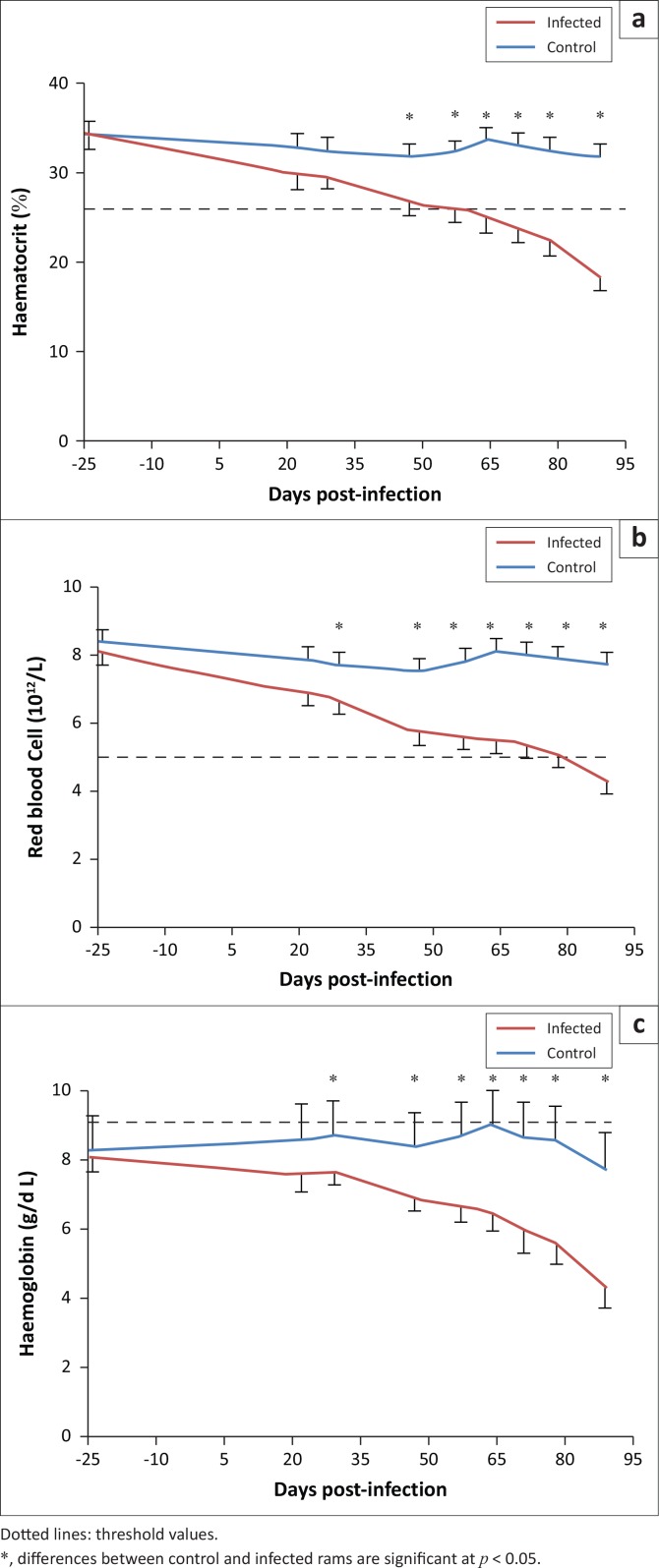
Variation of (a) haematocrit, (b) red blood cell count and (c) haemoglobin in *Haemonchus contortus* infected and control rams.

Factorial ANOVA with two independent factors showed a statistically significant effect of time, infection and the interaction infection*time on haematological parameters (*p* < 0.001). Repeated-measures ANOVA showed that the effect of time appears in infected group from day 47 post-infection. In fact, haematocrit, red blood cell count and haemoglobin decreased significantly in infected rams from 26.90% ± 1.06%, 5.69 ± 0.31 ×10^12^/L and 6.83 g/dL ± 0.29 g/dL at 47 days post-infection to 18.37% ± 1.81%, 4.24 ± 0.31 ×10^12^/L and 4.25 g/dL ± 0.46 g/dL, respectively (*p* < 0.05) at 89 days after infection (Figure 1a–c); corresponding values in control animals remained roughly constant (*p* > 0.05). The effect of infection was different for the three parameters. In fact, differences between control and infected rams started to be statistically significant from day 47 post-infection for haematocrit and from day 29 post-infection till the end of the trial for red blood cells and haemoglobin.

### Biochemical and clinical parameters

**FIGURE 2:**
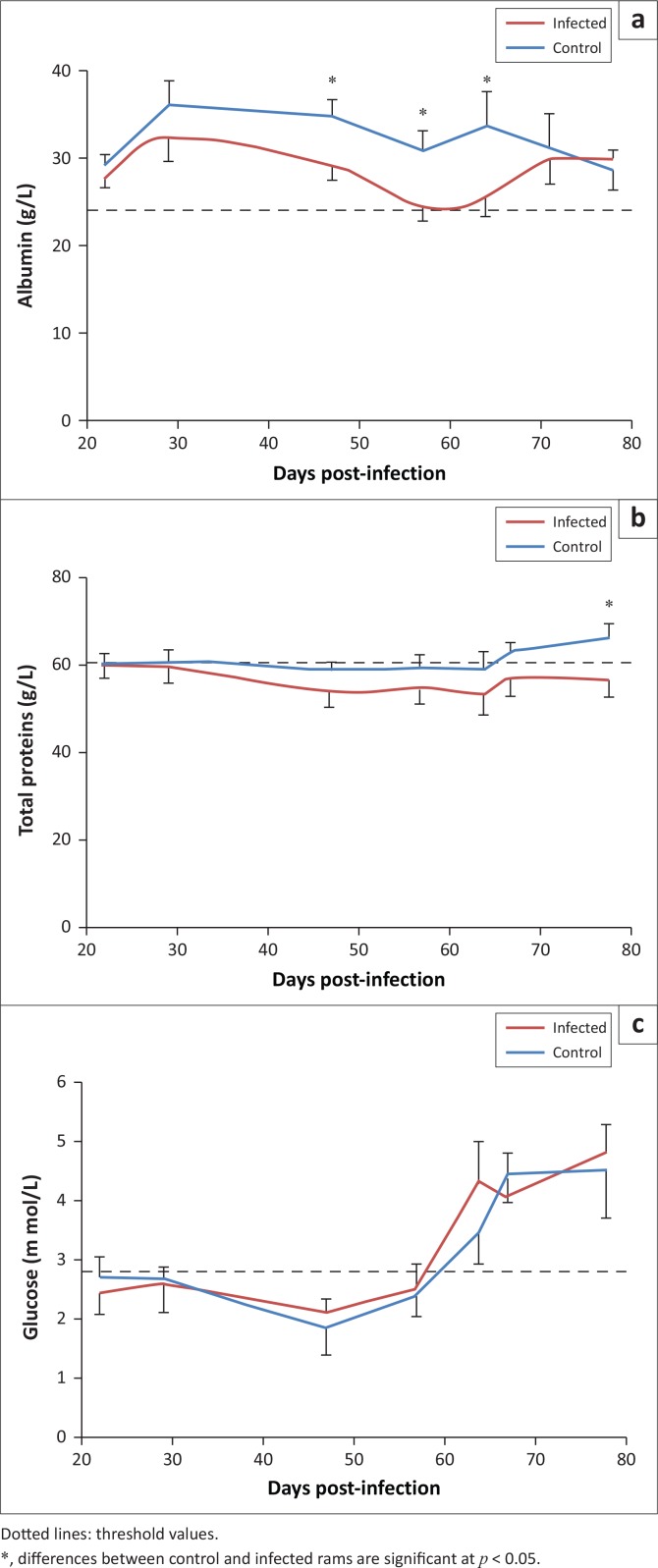
Variation of (a) albumin, (b) total proteins and (c) glucose in *Haemonchus contortus* infected and control rams.

Factorial ANOVA with two independent factors showed a statistically significant effect of time (*p* = 0.044) and infection (*p* = 0.002) for albumin. For total proteins, only treatment had a statistically significant effect (*p* = 0.003). For glucose, only time had a statistically significant effect (*p* < 0.001). For albumin and total protein concentrations, further analyses of the effect of infection showed that these concentrations were significantly lower in infected rams compared to control animals (*p* < 0.05). This difference was observed at days 47, 57 and 64 for albumin and at day 78 for total proteins (*p* < 0.05) (Figure 2a and b). Regardless of the infection status, the concentration of glucose increased in time (Figure 2c). No significant treatment differences were found for heart rate, respiratory rate frequency and rectal temperature.

### Live weight and scrotal circumference

Factorial ANOVA with two independent factors showed that there was not a statistically significant effect of infection, time and the interaction infection*time on live weight and scrotal circumference between infected and control animals (*p* > 0.05). Live weight averages were 61.75 kg ± 3.51 kg and 56.63 kg ± 4.59 kg at the beginning of the experiment and reached 65.58 kg ± 3.67 kg and 61.40 kg ± 2.26 kg at day 78 post-infection for control and infected rams, respectively. Scrotal circumference values amounted to 24.50 cm ± 1.85 cm and 26.67 cm ± 1.17 cm at day 22 post-infection to 24.58 cm ± 1.34 cm and 25.17 cm ± 1.73 cm at day 78 for control and infected animals, respectively.

### Semen traits

**FIGURE 3:**
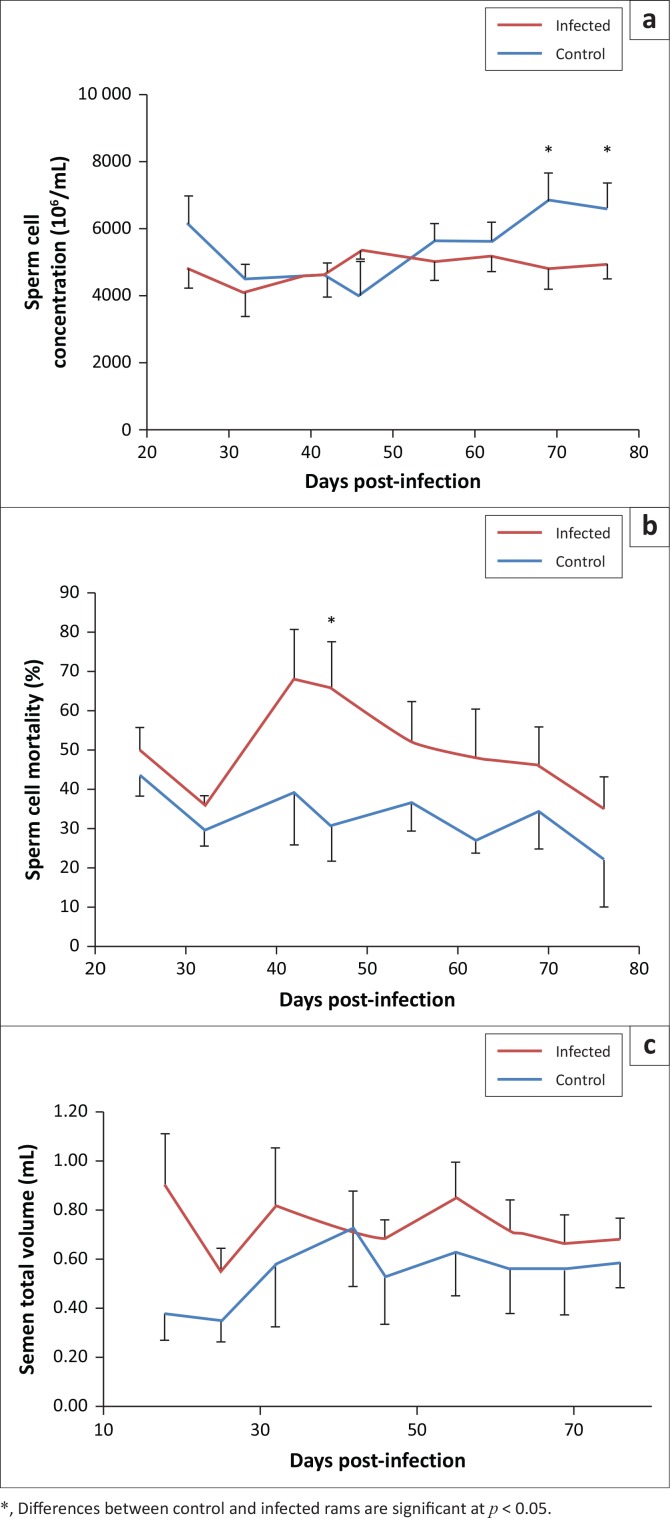
Variation of (a) sperm cell concentration, (b) sperm mortality rate and (e) ejaculate total volume in *Haemonchus contortus* infected and control rams.

Factorial ANOVA with two independent factors showed a statistically significant effect of infection and time for sperm concentrations (*p* = 0.016 and *p* = 0.032, respectively) and sperm mortality (*p* = 0.001 and *p* = 0.022, respectively). The effect of infection appears in time for sperm concentrations at days 69 and 76 post-infection (Figure 3a). Sperm mortality rate was significantly higher in infected animals (*p* < 0.05) at day 46 post-infection when compared to the control (Figure 3b) and tended to remain higher until the end of the experiment without reaching statistical significance. There was only a significant effect of infection on ejaculate volume (*p* = 0.04), but this effect was not observed for time (Figure 3c). Average figures were 0.55 mL ± 0.17 mL and 0.73 mL ± 0.14 mL for control and infected groups, respectively.

### Hormone concentrations

**FIGURE 4:**
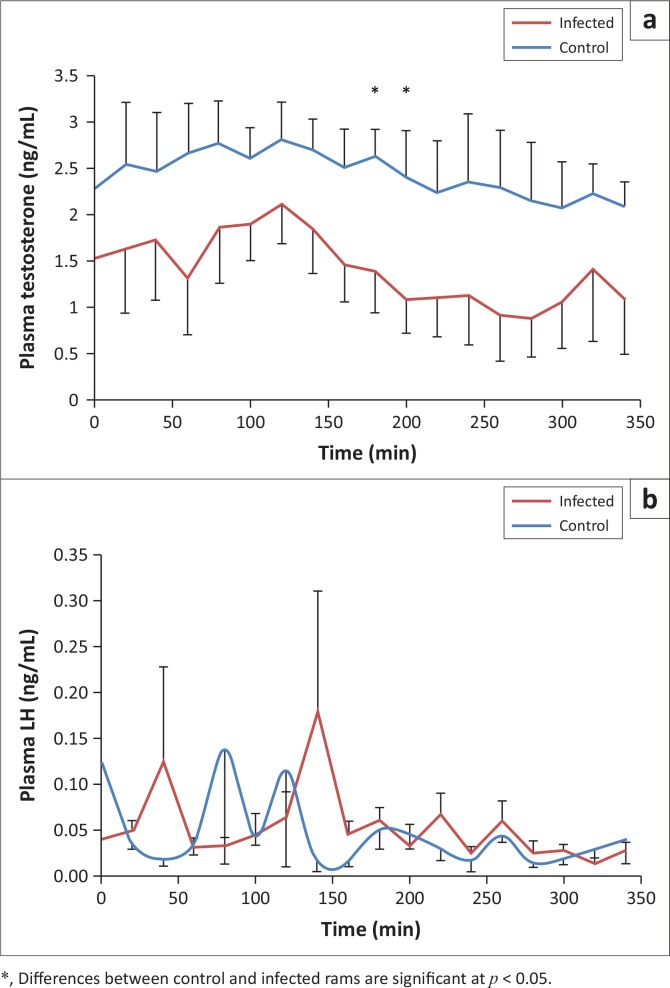
Variation of (a) plasma testosterone and (b) plasma luteinising hormone concentrations in *Haemonchus contortus* infected and control rams.

**FIGURE 5:**
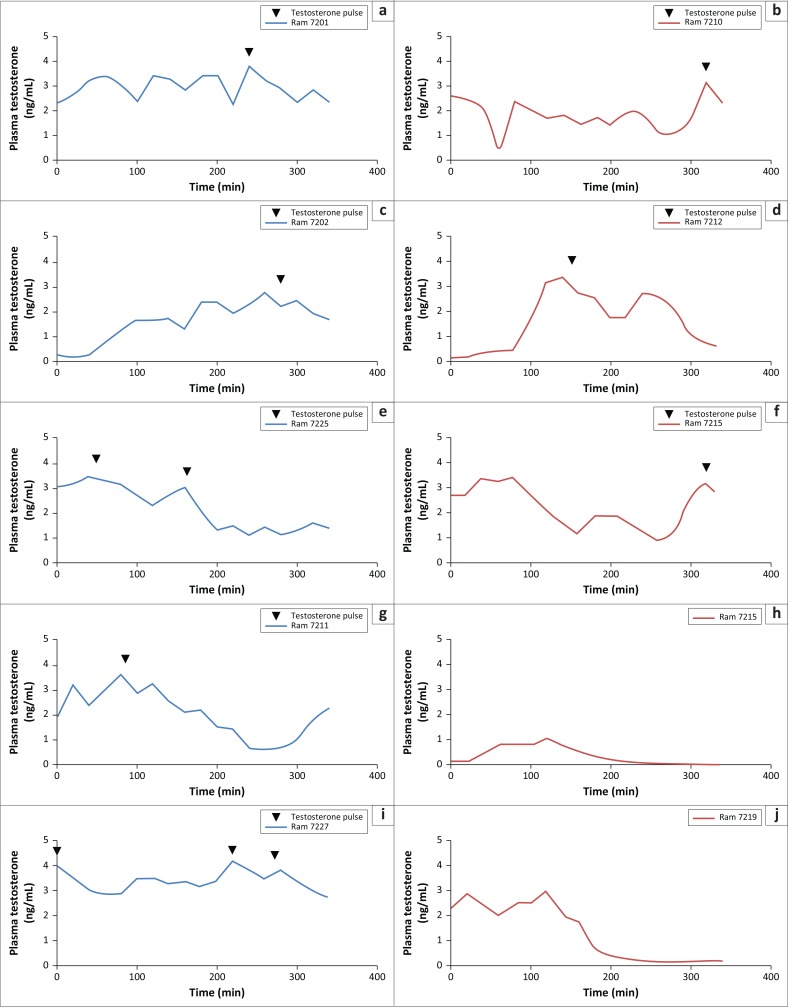
Plasma testosterone profiles in *Haemonchus contortus*. Control rams: (a) Ram 7201, (c) Ram 7202, (e) Ram 7225, (g) Ram 7211, (i) Ram 7227 and infected rams: (b) Ram 7210, (d) Ram 7212, (f) Ram 7215, (h) Ram 7216, (j), Ram 7219.

There was only a significant effect of infection on plasma testosterone concentrations (*p* < 0.001). Time and the interaction infection*time had no significant effect on plasma testosterone concentrations (*p* > 0.05). Overall the serial sampling period, mean concentrations of plasma testosterone concentrations were lower for infected than for control animals; significant differences were reached at the 10th and 11th sampling times. The same concentrations tended (0.08 < *p* < 0.1) to be lower at the 9th, 14th and 15th sampling times for infected versus control rams (Figure 4a). Testosterone pulse frequency tended (*p* = 0.06) to be lower in infected versus control rams (1.01 ± 0.12 and 1.42± 0.13, respectively) (Figure 5). For the individual testosterone profiles, areas delineated by measured concentrations were calculated using the trapezoid rule (Figure 5), and average figures were 479.28 ng/mL (SEM = 97.79) and 831.44 ng/mL (SEM = 107.22) for infected and control rams, respectively (*p* < 0.001). No significant effect of infection, time and the interaction infection*time was observed in plasma LH levels during the whole sampling period (*p* > 0.05) (Figure 4b). For control animals, there was a positive correlation between testosterone and LH plasma levels (*p* < 0.05); no such relationship was observed for infected animals.

## Discussion

Sheep are amongst the major economically important livestock species in Tunisia. Barbarine sheep is the most frequent breed representing 60% of the total sheep population in Tunisia, and it is also dominant in other North African countries such as Libya and Eastern Algeria (Ben Salem, Lassoued & Rekik 2011; Majdoub-Mathlouthi *et al*. 2013). The Barbarine breed is facing several environmental stressors. The major constraint is feed availability from natural pasture, which is quantitatively limited and has a very poor quality (Majdoub-Mathlouthi *et al*. 2013). In addition to this, the breed is also highly exposed to several health issues (Gharbi *et al*. 2013; Rjeibi *et al*. 2014) involving viral, bacterial and parasitic diseases such as toxoplasmosis (Khayeche *et al*. 2014), mange (Ben Chakroun & Jemli 1999), lungworms (Lahmar *et al*. 2013) and gastrointestinal parasites (Akkari *et al*. 2012). Gastrointestinal nematodes are amongst the most important pathogens of ruminants worldwide (Mahieu *et al*. 2009; Silva *et al*. 2015), and production losses caused by helminth species can be substantial (Albers *et al*. 1987; Beck, Moir & Meppem 1985). *Haemonchus contortus* is the main hematophagous gastrointestinal helminth of sheep; its high host specificity was confirmed by Silva *et al*. (2015).

The infected group received 30 000 *H. contortus* third-stage larvae orally, and at days 30 and 70 post-infection, the faecal egg counts in the six infected animals reached an average of 6200 eggs/g ± 664.24 eggs/g and 5000 eggs/g ± 886.00 eggs/g, respectively. Under other conditions, investigators reached different levels of haemonchosis using experimental infection (Getachew *et al*. 2015; Machado *et al*. 2014).

Some authors compared cryopreserved and unfrozen L3 of *H. contortus* infection (Van Wyk 1999). A mean of 33.4% of cryopreserved *H. contortus* L3 developed in sheep, compared with a percentage of 43.7 for unfrozen L3 of the same species.

Results from this study confirmed that infection had actually occurred since *H. contortus* infection is known to cause significant changes of haematological parameters mainly anaemia (Al-Quaisy *et al*. 1987). This is reflected by the significant decrease in haemoglobin, red blood cells count and haematocrit in response to *H. contortus* infection. It was also established that there is a gradual decline in haemoglobin concentration during the post-infection period (Bordoloi *et al*. 2012), red cell losses (Dargie & Allonby 1975) and reduction of the haematocrit (Albers *et al*. 1990).

The main objective of this work was to test the effect of *H. contortus* infection on reproductive traits in adult rams. Although *H. contortus* infections are a well-known problem, to our knowledge there are few published studies dealing with the effect of *H. contortus* infection on semen characteristics in sheep. In our study, semen traits were depressed due to the infection. All changes took place towards the middle, up to the end of the experiment. These changes cannot be solely related to the animal’s health status since infected animals did not show modification in clinical and body condition parameters after infection with *H. contortus*. In fact, live weight, heart rate, respiratory rate and rectal temperature averages did not show major differences between infected and control rams; this is one more indication of the adaptive capacity of the studied breed to cope with harsh environmental conditions and non-optimal husbandry conditions (Ben Salem *et al*. 2011). Our finding is not consistent with other reports. Indeed, Hayat *et al*. (1996) found that inhabiting the abomasum of sheep and goats, *H. contortus* cause blood loss resulting in reduced feed intake and decreased body weight. Recently, Tonin *et al*. (2014) reported that crossbred Corriedale lambs infected with 15 000 third-stage larvae of *H. contortus* given as three divided doses were losing their physiological condition progressively; getting weak, lethargic, pale and experiencing depressed feed intake. Further, subclinical parasitism in dairy goats resulted in a reduction in body condition score when infected with 5000 third-stage larvae (Hoste & Chartier 1993).

In this study, infected animals presented a decrease in plasma testosterone concentrations compared to control rams. Testosterone levels after 4 weeks of infection with 5000 infective larvae of *H. contortus* were shown to have a significant positive correlation with worm burden (Gauly *et al*. 2006). It was concluded in the same study that female lambs were more resistant to an experimental *H. contortus* infection when compared with male lambs, and testosterone seemed to play an important role in resistance. In our study, the modification in testosterone concentrations was not accompanied by a change in LH concentrations. This shows that the function of the hypothalamus-pituitary system was not affected by the infection. Changes in plasma testosterone concentrations may be due to a disruption taking place in the Leydig cells’ function, hampering their response to LH stimulus. The lack of a correlation between LH and testosterone plasma levels in infected animals supports this statement. However, this working hypothesis requires further experimental evidence.

Nevertheless, other workers did not show an effect of parasite infection on reproductive performances of Santa Inês and Morada Nova ewes in Brazil. Females of these two breeds showed good reproductive performance, with no differences in conception rate, return to oestrus, multiple births and prolificacy (Issakowicz *et al*. 2016). A differentiated sex response to infection with *Haemonchus* may therefore be suspected between these results from Brazil and our findings.

## Conclusion

Though preliminary, these results are important as far as the management of sheep reproduction with relation to parasite infection under conditions of insufficient health care is concerned. Significant changes were noticed between infected and control animals for reproductive parameters. The study could not determine whether *Haemonchus*-induced anaemia was the primary cause compromising male reproduction or whether infection directly affected sperm traits. It is also important from a management perspective to assess whether the recorded depression in reproductive traits has an impact on the actual mating capacity of the rams under field conditions.
